# Expectation framing in psychotherapy: effects of approach- and avoidance-oriented video interventions on help-seeking intentions

**DOI:** 10.3389/fpsyt.2026.1869832

**Published:** 2026-07-09

**Authors:** Leonora N. Schäfer, Winfried Rief

**Affiliations:** Department for Clinical Psychology and Psychotherapy, Philipps University Marburg, Marburg, Germany

**Keywords:** expectancy effects, help-seeking intentions, message framing, psychotherapy engagement, role-model intervention, social anxiety, treatment expectations

## Abstract

**Introduction:**

Treatment expectations play an important role in psychotherapy initiation and early treatment engagement may influence whether individuals decide to seek help. Negative expectations, concerns about burden, and anticipated risks may be particularly relevant for individuals with social anxiety. However, little is known about how different ways of framing treatment expectations influence help-seeking intentions. This study examined whether brief, role-model-based video interventions can modify treatment expectations and increase the subjective likelihood of seeking psychotherapy.

**Materials and methods:**

Participants (N = 123) were randomly assigned to one of three conditions: (1) avoidance-oriented framing, emphasizing the reduction of negative treatment expectations; (2) approach-oriented framing, highlighting potential positive treatment outcomes or (3) a minimal-intervention control condition. Treatment expectations (TEX-Q) and self-reported likelihood of seeking psychotherapy were assessed before and after the intervention.

**Results:**

Both video conditions increased subjective treatment-seeking probability compared to the control group, with a stronger increase observed for avoidance-oriented framing. This pattern was replicated in a subsample with higher self-reported social anxiety. Effects on treatment expectations were less consistent.

**Conclusion:**

These findings suggest that expectation-focused communication may help address expectation-related barriers before psychotherapy begins. Avoidance-oriented framing may be particularly relevant in the early phase of treatment engagement, especially for individuals with higher self-reported social anxiety.

**Trial registration:**

The trial was registered at ClinicalTrials.gov: NCT06022432.

## 1 Introduction

Numerous studies have shown that, despite high mental health needs, there is a significant gap between the demand for and utilization of psychotherapy (1–3). Although psychological treatments are widely accepted and strongly supported by empirical evidence (4–6), help-seeking remains relatively low, particularly among individuals with social anxiety. Many socially anxious individuals report delayed treatment initiation or no professional treatment at all (7–9). Given the considerable burden and comorbidity associated with social anxiety, improving access to and engagement with psychotherapy remains an important clinical goal (10–13).

Barriers to treatment-seeking in social anxiety include structural factors such as long waiting times, costs, limited access to specialized care, and low awareness of available services, as well as attitudinal barriers such as stigma, embarrassment, and low perceived need for treatment (14–16). While structural barriers require systemic changes, attitudinal barriers reflect internal beliefs and expectations that directly shape help-seeking decisions. In social anxiety, fear of negative evaluation and self-stigma may further amplify these psychological barriers. Accordingly, targeting modifiable psychological factors such as treatment expectations may be a promising starting point for supporting psychotherapy initiation and early engagement (17).

Expectations represent a central psychological mechanism linking beliefs about psychotherapy to actual help-seeking behavior. According to expectancy models, anticipated benefits and concerns influence treatment decisions, adherence, and clinical outcomes (18, 19). In social anxiety, negatively biased interpretations and avoidance-oriented cognitive styles may foster unfavorable expectations about psychotherapy (20). Empirical studies further indicate that lower perceived need and less favorable attitudes toward treatment are associated with delayed initiation or non-use of mental health services (21, 22). As expectations can be modified relatively easily, they may represent a low-threshold target for pre-treatment interventions aimed at facilitating psychotherapy initiation. From a health psychology perspective, help-seeking intentions can be understood as proximal indicators of later treatment-seeking behavior. This idea is also reflected in the Theory of Planned Behavior, which assumes that intentions are an important predictor of behavior (23). However, intentions do not always translate into action and the association between intention and actual behavior is often only moderate. The intention-behavior gap is particularly important in the context of psychotherapy initiation, where additional barriers such as stigma, avoidance and structural obstacles may prevent individuals from acting on their intentions (24). In this study, treatment expectations can be understood as expectations about possible benefits and barriers of psychotherapy. Such benefits and barriers are also important in health-behavior models, for example in the Health Belief Model (25). According to this model, health-related action becomes more likely when individuals perceive benefits of an action and fewer barriers to performing it. Applied to psychotherapy, positive treatment expectations may increase perceived benefits, whereas negative expectations may function as perceived barriers to seeking help. Thus, although this study is mainly based on psychotherapy-expectancy research, health psychology offers an additional perspective on how expectations may influence early engagement with psychotherapy.

One communication strategy that can systematically shape expectations is message framing. Framing refers to presenting identical information in either a gain-focused or loss-focused manner, thereby influencing risk perception and decision-making (26, 27). For example, in medical decision-making, presenting identical statistics in terms of survival versus mortality can shift patients’ evaluations and willingness to undergo medical procedures (28). In addition, recent research indicates that subtle differences in how probabilistic information is communicated can systematically influence expectations about treatment outcomes and perceived risks (29, 30). In the context of psychotherapy, framing can be understood as focusing either on expected benefits (approach-oriented framing) or on reducing potential burdens and risks (avoidance-oriented framing). While this distinction is related to gain- and loss-framing, the present study adopts a motivational perspective, distinguishing between approach- and avoidance-oriented expectation framing. Individuals with higher trait anxiety appear to be more influenced by such framing effects, particularly when information emphasizes potential losses or risks (31, 32). Beyond immediate decision-making, framing can also influence treatment-related expectations. According to the expectancy model, expectations influence symptom perception, side-effect reporting, and treatment outcomes (18, 19). Importantly, positive and negative treatment expectations represent partially distinct dimensions and are only modestly correlated, allowing individuals to simultaneously anticipate benefits and fear adverse outcomes (33). For example, a person may believe that psychotherapy could help them reduce symptoms and improve daily functioning, while at the same time worrying that therapy could be stressful or difficult to start. Empirical evidence further shows that modifying the framing of side-effect information can alter these expectations: Positively framed information has been associated with reduced nocebo-related symptoms and lower negative expectations compared to standard wording (34). By shaping anticipatory beliefs, framing interventions may therefore reduce expectation-related barriers and facilitate help-seeking, which may be particularly relevant for individuals with social anxiety.

The present study examined whether brief, low-threshold online interventions can modify treatment expectations and increase the subjective likelihood of seeking psychotherapy as a proximal indicator of help-seeking intention. Specifically, we compared two expectation-framing approaches: first, avoidance-oriented framing, emphasizing the reduction of negative treatment expectations, and secondly, approach-oriented framing, highlighting potential positive treatment outcomes. These conditions were compared to a minimal-intervention control group, in which participants described their current treatment expectations. We further investigated whether the two framing approaches would produce differential effects on treatment expectations and help-seeking intentions, and whether these effects varied as a function of self-reported social anxiety. Outcomes included changes in treatment expectations and the subjective likelihood of seeking psychotherapy.

We hypothesized that participants receiving a framing-based expectancy intervention would show greater improvements in treatment expectations and help-seeking intentions compared to the control group. In addition, we examined whether the two framing orientations would lead to differential effects, suggesting distinct underlying mechanisms. Finally, we explored whether individuals with higher levels of social anxiety would respond differently to the two framing approaches.

## 2 Materials and methods

### 2.1 Participants

An *a priori* power analysis using G*Power (Version 3.1) was conducted to determine the required sample size. A sample size of N = 111 was needed to detect a small effect (*f* = 0.15) with a power of .80 and an alpha level of *α* = .05, based on the omnibus Time × Condition interaction in a repeated-measures ANOVA with three groups and two time points. We aimed to recruit a surplus of participants in case of exclusions based on pre-established criteria. Planned contrasts were part of the primary analyses but were not powered separately. The subgroup analysis was considered secondary.

Participants were informed about the study through university mailing lists, public postings in the city center, social media channels (Facebook and Instagram), and mailing lists of nationwide self-help groups focusing on social anxiety. Recruitment took place from March 2023 to February 2024. The recruitment materials explicitly addressed individuals who experienced social anxiety. Participants entered the study by self-selection, without a formal diagnostic assessment or predefined LSAS-SR cutoff at intake. Social anxiety symptoms were assessed during the study using the LSAS-SR. Participants had to be at least 18 years old, fluent in German and have access to an internet-enabled device suitable for completing online questionnaires and viewing video material. Further exclusion criteria were significant visual or hearing impairment (if not corrected), current psychotherapeutic treatment or registration on a waiting list and neurological impairment or psychotic disorders. A self-reported prior or current mental disorder diagnosis and prior treatment experience were not exclusion criteria, provided that participants were not currently receiving psychotherapy and were not registered on a waiting list. The final sample was composed of 123 participants with different levels of previous contact with mental health care but without current psychotherapeutic treatment. As an incentive, participants had the chance to win one out of eight online vouchers valued at €25. The study was reviewed and approved by the Ethics Committee of the Department of Psychology at the University of Marburg (reference number: 2022-76k) and conducted in accordance with the ethical guidelines of the German Psychological Society. All participants gave written informed consent.

### 2.2 Treatment rationale

Two experimental groups received a video intervention of a reenacted patient-therapist interaction. In both videos, a female ‘patient’ reports to her therapist about her initial concerns, treatment expectations and overall positive experience during the treatment-seeking and therapy process. Both videos were approximately six minutes long and followed the same thematic sequence: a brief greeting/introduction, a discussion of treatment expectations before seeking therapy, the treatment-seeking process and associated expectations or concerns, the start of therapy and the therapy process including perceived treatment effects and experienced changes. The therapist’s verbal statements were identical across conditions, and only the patient’s statements differed according to the respective framing condition. These patient statements were inserted into otherwise identical video material. Thus, the setting, dyad, sequence of the conversation, and overall video length were kept constant across both intervention conditions. Participants were blinded to the fact that they were watching one of two highly similar videos of a patient-therapist interaction which differed only in the framing of treatment expectations and outcomes.

Participants in the ‘Reducing Negative Expectations’ group (RN-EXP) watched a video in which the patient’s testimonial was framed to reduce negative expectations about psychotherapy. In the video, the patient’s testimonial featured statements such as ‘I *became less insecure and increasingly less unsure* about how to express myself, I became *less hesitant* and with each additional meeting, *it became less difficult* for me to come here’ or ‘The more often I confronted my fears, *the less difficult it became* over time, ultimately leading to *less hesitation and self-doubt* in social situations.’. In the ‘Maximizing Positive Expectations’ condition (MP-EXP) the patient’s testimonial was framed to maximize positive treatment expectations. The positively worded statements of the patient included phrases like ‘*I became more confident and increasingly clear* in expressing myself’, ‘I became *more courageous* and with each additional meeting, *it became easier* for me to come here’, ‘The more often I confronted my fears, *the easier it became* over time, ultimately leading to *greater confidence and self-assurance* in social situations’.

To provide a comparison condition, the control group was assigned to describe their current treatment expectations and possible apprehensions about seeking treatment in a free-writing task.

### 2.3 Task engagement

Participants who received a video intervention (RN-EXP and MP-EXP) had to answer follow-up questions regarding their task engagement. They were asked whether they attentively watched the video, felt distracted during the intervention, and were able to follow the conversation. Responses were given using the categories ‘yes’, ‘no’ and ‘partially’. Participants who reported insufficient engagement with the task were excluded from further analyses.

To ensure credible task engagement of the control group in the free-writing task, only participants were included whose responses demonstrated at least a minimal level of meaningful engagement with the task. This was operationalized as written input that extended beyond a single character and was thematically relevant, logically structured and comprehensible.

### 2.4 Experimental procedure

The study was conducted online, and participants completed all procedures independently at a time of their choosing, without live contact with study investigators. The survey could be accessed via computer, tablet, or smartphone. However, participants were advised to use a device with stable internet access and adequate audio-visual quality because the intervention included video material. At the beginning of the online procedure, participants were informed that the study aimed to investigate treatment barriers, the estimated efficacy of therapeutic treatment for social anxiety and associated attitudes. They were also informed that they would be invited to complete a one-week follow-up assessment. After giving written informed consent, participants were randomly assigned to one of the three experimental groups and completed questionnaires assessing baseline measures. Participants of the RN-EXP and MP-EXP conditions were told that they would watch a pre-recorded video of a patient-therapist interaction. The videos differed in verbal framing about treatment expectations (see 2.2). Instead of receiving a video intervention, participants of the control group were asked to write freely about their expectations and apprehensions regarding therapeutic treatment. Subsequently, treatment expectations and subjective likelihood of seeking treatment were measured again, and participants could indicate whether they were interested in receiving an email with detailed information and links to publicly accessible information and contact pages about social anxiety and its evidence-based treatment options. One week later, all randomized participants who had completed the main assessment were invited by email to complete the follow-up assessment. To link follow-up responses to baseline and post-intervention data, participants were asked to use the same individualized code they had created during the main assessment. Of the 171 participants invited to participate in the follow-up 35 completed the follow-up assessment. However, only 26 follow-up responses could be reliably linked to the main assessment because some participants used different or incorrect codes. Due to the small, linked follow-up sample and very small group sizes, follow-up data were analyzed descriptively only. Participants were subsequently debriefed about the study design and its aims.

**Figure 1:**
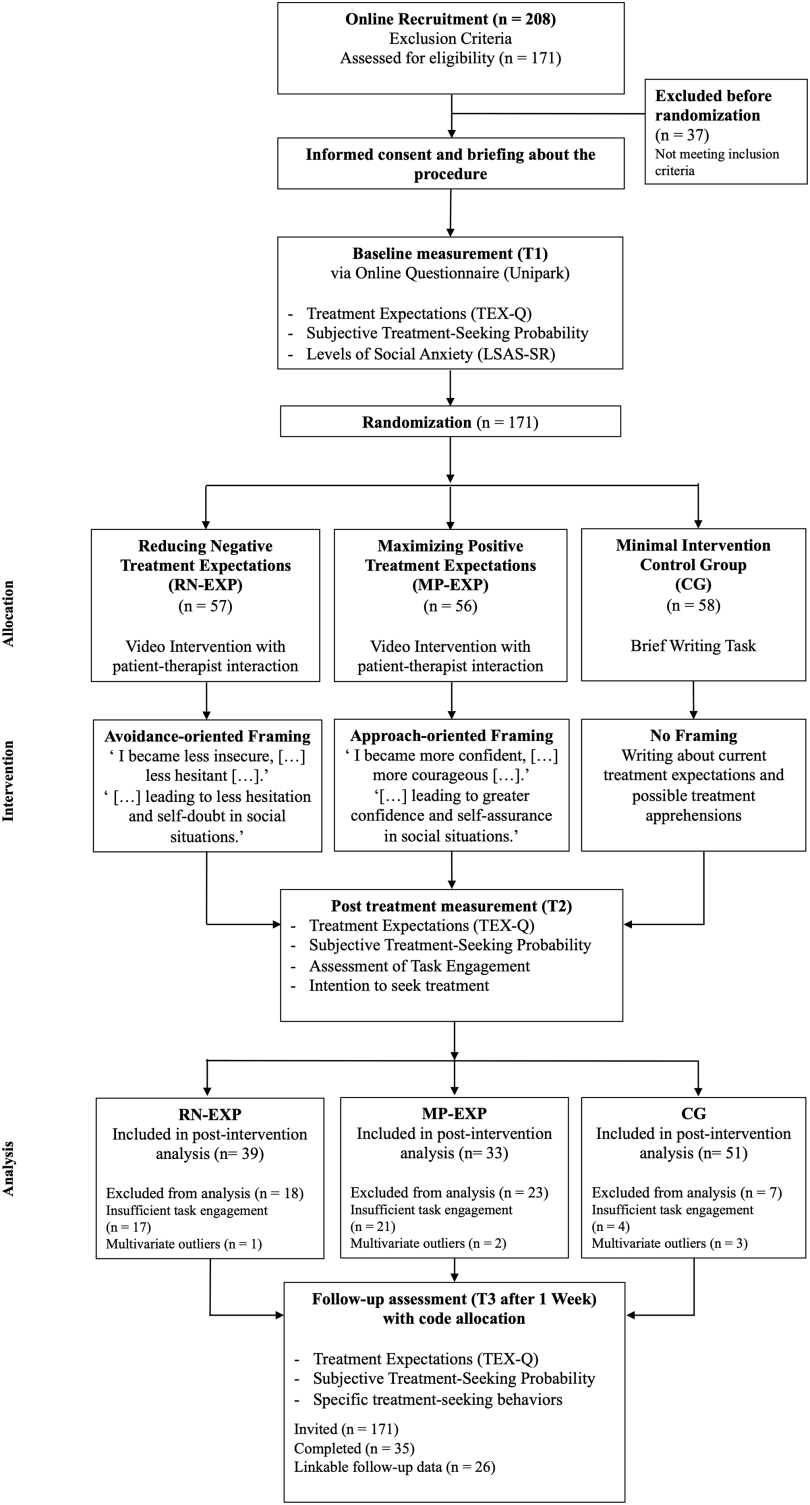
Participant flow and study procedure. All participants completed a baseline assessment (T1) and were then randomly assigned to one of three conditions: reducing negative treatment expectations (RN-EXP), maximizing positive treatment expectations (MP-EXP), or a minimal-intervention control group (CG). After the intervention (video-based framing in RN-EXP and MP-EXP; brief writing task in CG), post-intervention measures were assessed (T2). The figure shows allocation, exclusions due to insufficient task engagement and multivariate outliers, and the number of participants included in the post-intervention analyses. A one-week follow-up was planned and completed by a smaller subsample; due to substantial attrition and data linkage limitations, follow-up data were not used for inferential group comparisons.

For more details, see **Figure 1**.

### 2.5 Measures

All questionnaires were administered online via the survey software Unipark (www.unipark.com), a web-based survey platform commonly used for online research.

#### 2.5.1 Treatment expectations

Explicit treatment expectations were measured by the German version of the Treatment Expectation Questionnaire ‘TEX-Q’ (35). This 15-item questionnaire measures patient expectations of medical and psychological treatments regarding treatment benefits, adverse events, negative and positive impact as well as process and behavioral control using an 11-point Likert scale. The TEX-Q total score was calculated by summing all 15 items. Items from negatively framed domains, including adverse effects and negative impact, were reverse coded before calculating the total score. Thus, higher total scores indicate more favorable treatment expectations, reflecting stronger positive expectations and lower negative expectations. The TEX-Q demonstrated good to excellent internal consistency, with Cronbach’s alpha values ranging from .71 to .92. In the present study the internal consistency was α = .91.

#### 2.5.2 Subjective treatment-seeking probability

The subjective likelihood of seeking psychotherapeutic treatment was used as an indicator of help-seeking intention. It was assessed using a self-developed rating scale ranging from 0 (‘not likely at all’) to 100 (‘very likely’), with higher values indicating a greater likelihood of seeking treatment.

#### 2.5.3 Self-reported behavioral indicators of treatment-seeking

Participants’ interest in treatment information was assessed by asking whether they wished to receive further information about social anxiety and available treatment options (‘yes’ or ‘no’). Participants who indicated interest could provide their email address to receive information about possible first steps toward treatment, including links to therapist search platforms, mental health websites, and self-help resources. Providing an email address was used as an initial behavioral indicator of movement toward psychotherapy initiation, but not as evidence of actual psychotherapy uptake.

At follow-up, participants were asked whether they had taken any self-reported steps toward seeking further information or support for social anxiety during the previous week. These steps included searching for additional information online, engaging with internet platforms or self-help groups, participating in online therapy programs or sessions, or contacting professional mental health services (e.g. outpatient therapist, outpatient clinics or psychiatric facilities). Responses for each behavior were recorded on a dichotomous scale (‘yes’ or ‘no’).

#### 2.5.4 Social anxiety

Social anxiety levels were assessed with the German version of the Liebowitz Social Anxiety Scale, Self-Report (LSAS-SR) (36). The LSAS-SR is a 24-item self-report measure that assesses subjectively experienced fear and avoidance of social situations during the past week. Each item is rated separately for fear and avoidance on a four-point Likert scale ranging from 0 to 3. The LSAS-SR shows excellent internal consistency (α = .95) and good test-retest reliability [r = .83; e.g. (37, 38)]. The internal consistency for the LSAS-SR in the present study was excellent (α = .95).

### 2.6 Statistical analysis

Because the study design required participants to enter all values before continuing, no missing values were reported. Univariate outliers of primary outcomes (treatment expectations and subjective treatment-seeking probability) were identified using standardized z-values and visual histogram inspection. To detect multivariate outliers the Minimum Covariance Determinant (MCD) method was used, which provides a robust estimation of Mahalanobis distances (39). Observations exceeding the χ²-based cut-off (97.5^th^ percentile) were classified as multivariate outliers and subsequently excluded to prevent violations of model assumptions (40–42).

To investigate whether the groups differed in baseline values of primary outcomes, a multivariate analysis of variance (MANOVA) was conducted with condition as the independent variable (IV) and baseline values of treatment expectations, subjective treatment-seeking probability, age and social anxiety levels as dependent variables (DVs). Using chi-square tests, the distribution of gender, educational level and employment status as well as self-reported current mental disorder status and prior treatment experience was examined between experimental groups.

Two separate mixed-effect ANOVAs were performed to analyze changes in outcomes of treatment expectations and subjective treatment-seeking probability with timepoint and condition as independent variables. *Post hoc* analyses with Bonferroni-Holm correction were performed using planned contrast coding. For the first contrast both treatment groups (RN-EXP and MP-EXP) were compared to the control group (−1, −1, 2) and for the second contrast both video intervention groups were compared with each other (−1, 1, 0). The primary analyses were conducted as per-protocol complete-case analyses including participants who completed the pre- and post-intervention assessments, met the predefined task engagement criteria and were not classified as multivariate outliers. To examine whether exclusions based on task engagement affected the results, modified intention-to-treat sensitivity analyses were additionally conducted including all participants with available pre- and post-intervention outcome data regardless of task engagement. Multivariate outliers remained excluded in these analyses. To address the potential influence of prior treatment experience, additional sensitivity analyses were conducted including prior treatment experience as a binary covariate. Prior treatment experience was coded as any previous outpatient psychotherapy, day-clinic treatment, or inpatient treatment.

To examine whether the effects were also present among participants with higher self-reported social anxiety, a secondary analysis with participants scoring 35 or higher on the LSAS-SR was conducted. As scores below 35 have been proposed as a remission threshold for social anxiety symptoms in a German sample, scores of 35 and higher were used here as an indicator of elevated self-reported social anxiety symptoms for subgroup analyses and not as a diagnostic criterion (43).

All analyses were performed using R 4.5.1 and RStudio 2025.05.1 + 513.

## 3 Results

### 3.1 Sample characteristics

For the study, a total of 208 participants were recruited. Of these, 171 participants fulfilled the inclusion criteria for this study. After checking for task engagement, a total of 42 participants were excluded from further analyses. Thirty-eight participants in the treatment groups (21 in MP-EXP and 17 in RN-EXP) reported that they had not attentively watched or followed the video intervention and four participants in the control group did not properly engage in the free-writing task. Six of the remaining participants in the study sample were identified as multivariate outliers for primary outcomes and were excluded from further analyses as well. Experimental groups were unequal in size, with 39 and 33 participants in the video intervention groups and 51 participants in the control condition.

A sensitivity analysis including multivariate outliers was performed. The results of the analysis indicated changes in the reported pattern of results for the outcome of treatment expectations (for further details, see Section 3.3.1.).

Participants ranged in age from 18 to 70 years old (*M* = 27.98, *SD* = 9.05). The sample included 95 women (77.24%), 25 men (20.33%) and 3 diverse participants (2.44%). The majority of the sample were university students (63.41%). Participants of the present study reported moderate levels of social anxiety (LSAS-SR Sum Score *M* = 70.24, *SD* = 26.50) according to von Glischinski, Willutzki, Stangier, Hiller, Hoyer, Leibing, Leichsenring and Hirschfeld (43). Overall, 50.4% of participants reported currently experiencing mental disorders and 76.42% of the sample reported at least partially experiencing treatment barriers.

**Table 1** presents demographic sample characteristics.

**Table 1:** Descriptive statistics of sample characteristics and baseline values.

Variables	RN (*N* = 39)	MP (*N* = 33)	CG (*N* = 51)
Age, M (SD)	28.31 (7.96)	27.24 (7.20)	28.22 (10.87)
LSAS Sum Score, M (SD)	69.41 (25.71)	74.06 (27.54)	68.41 (26.69)
Gender, N (%)			
Male Female Diverse	9 (23.08) 29 (74.36) 1 (2.56)	7 (21.21) 25 (75.76) 1 (3.03)	9 (17.65) 41 (80.39) 1 (1.96)
Educational Level, *N (%)*			
School student Secondary education High school degree University degree	1 (2.56) 2 (5.13) 21 (53.85) 15 (38.46)	0 (0) 5 (15.15) 18 (54.55) 10 (30.30)	0 (0) 4 (7.84) 28 (54.90) 19 (37.26)
Profession, *N (%)*			
Student Employee	21 (53.85) 18 (46.15)	22 (66.67) 11 (33.33)	35 (68.63) 16 (31.37)
Treatment Expectations T1, *M (SD)*	97.90 (22.87)	99.91 (22.48)	100.41 (25.91)
Subjective Treatment-Seeking Probability, *M (SD)*	49.23 (29.88)	54.70 (35.16)	53.14 (31.45)
Experience of treatment barriers			
Yes Partially No	12 (30.77) 19 (48.72) 8 (20.51)	14 (42.42) 12 (36.36) 7 (21.21)	18 (35.29) 19 (37.26) 14 (27.45)
Current experience of Mental Disorder, *N (%)*			
Yes (Officially diagnosed) Yes (Not officially diagnosed) No Social Anxiety Disorder Depressive Disorder Other Anxiety Disorders Personality Disorders AD(H)D Others	15 (38.46) 7 (17.95) 17 (43.59) 7 (17.95) 13 (33.33) 4 (10.26) 4 (10.26) 2 (5.13) 3 (7.69)	8 (24.24) 8 (24.24) 17 (51.51) 7 (21.21) 8 (24.24) 6 (18.18) 11 (33.33) 1 (3.03) 1 (3.03)	17 (33.33) 7 (13.73) 27 (52.94) 10 (19.61) 14 (27.45) 7 (13.73) 2 (3.92) 2 (3.92) 1 (1.96)
Prior Treatment Experience, *N (%)*			
Previous Outpatient Therapy Previous Day-clinic Treatment Previous Inpatient Treatment	19 (48.72) 3 (7.69) 4 (10.26)	9 (27.27) 0 (0) 3 (9.09)	20 (39.22) 5 (9.80) 6 (11.76)

‘Officially diagnosed’ refers to a diagnosis made by a healthcare professional. ‘Not officially diagnosed’ refers to participants’ self-reported suspicion or experience of a mental disorder without formal diagnosis. Multiple responses were possible for mental disorder categories and prior treatment experience. M, Mean; SD, Standard deviation; N, Number of participants, % = Percentage of participants.

### 3.2 Examination of baseline differences

Given the unequal group sizes, the Box’s M test was conducted to assess the assumption of homogeneity of covariance matrices. The test was non-significant (*p* = .078) indicating that the assumption was met. Results of the MANOVA indicated no significant differences in baseline values between the experimental groups (*Pillai’s* Trace = .015, *F*(8, 236) = 0.228, *p* = .986, partial η² = .008). The distribution of gender was not significantly different across the three groups (*χ²*(4) = 0.553, *p* = .968, *Cramér’s V = .*07.), nor were the distributions of educational level (*χ^2^*(8) = 8.191, *p* = .415, *Cramér’s V* = .26) or employment status (*χ^2^(2)* = 2.287, *p* = .319, *Cramér’s V* = .14.). The distribution of self-reported current mental disorder status (*χ^2^*(4) = 2.791, *p* = .593, *Cramér’s V* = .15) as well as prior treatment experience did not differ significantly between conditions (*χ^2^*(2) = 2.523, *p* = .283, *Cramér’s V* = .14).

### 3.3 Main analysis

#### 3.3.1 Treatment expectations

**Figure 2:**
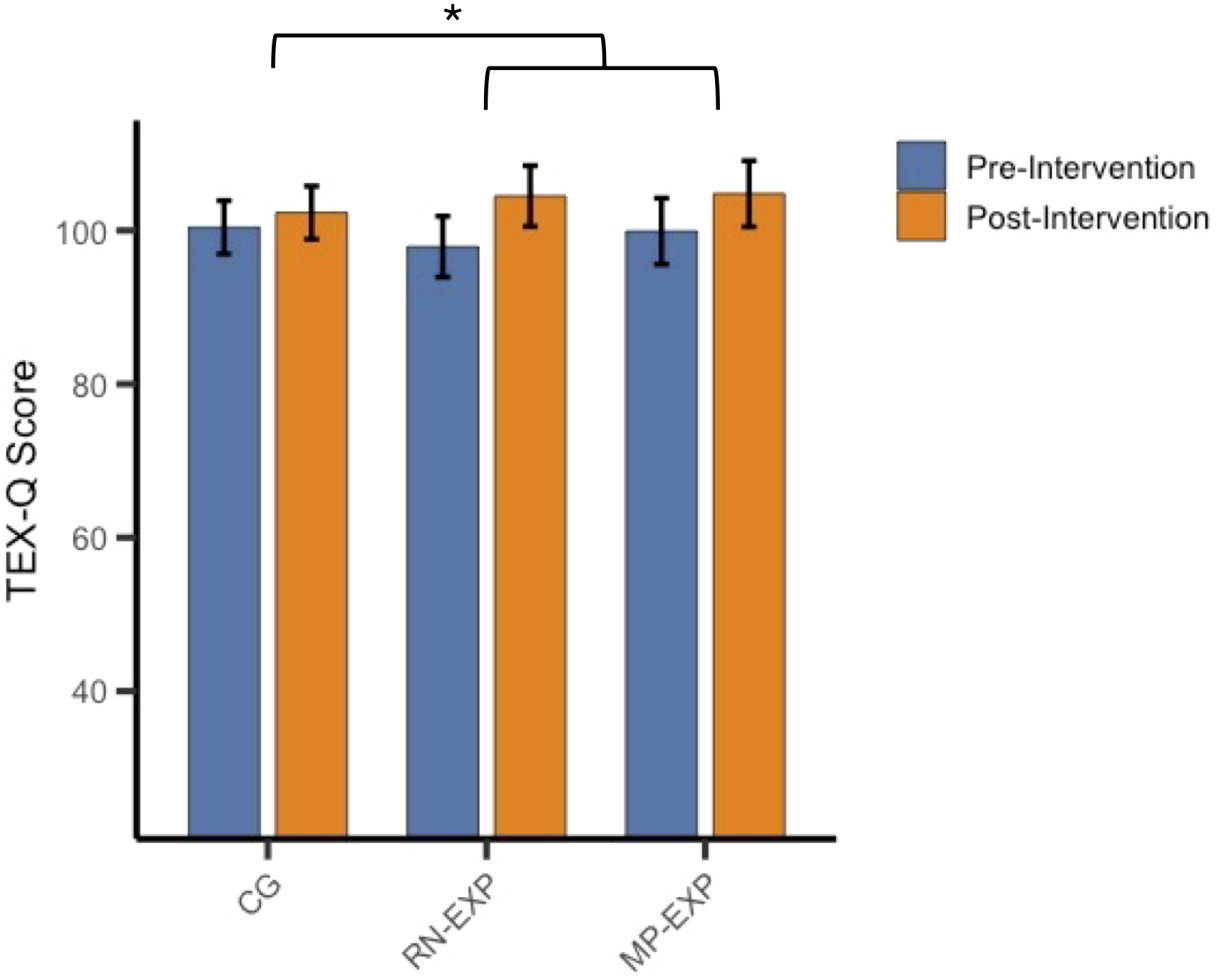
Change in treatment expectations. Planned contrasts showed that participants in both video intervention groups (RN-EXP and MP-EXP; patient-therapist interaction) reported a larger increase in treatment expectations than the minimal-intervention control group. The two video intervention groups did not differ significantly in expectation change. Note. Error bars indicate standard errors. Descriptive values are reported as M (SD): RN-EXP pre = 97.90 (22.87), post = 104.49 (24.29); MP-EXP pre = 99.91 (22.48), post = 104.79 (25.32); CG pre = 100.41 (25.91), post = 102.32 (26.19). CG = minimal-intervention control group (brief writing task about treatment expectations); RN-EXP video intervention with avoidance-oriented framing to reduce negative treatment expectations; MP-EXP = video intervention with approach-oriented framing to increase positive treatment expectations. *p<.05.

Results of the mixed-effects ANOVA indicated that the reported increase in treatment expectations after the intervention phase differed significantly between the experimental groups (Time × Condition: *F*(2,120) = 3.076, *p* = .049, *ω^2^* = .03). Planned contrasts indicated a stronger increase of treatment expectations for both video intervention groups in comparison to the control group (*b* = 3.832, *SE* = 1.664, 95% CI = [0.55, 7.11], *p = .*023, *ω^2^* = .04). However, no significant differences emerged between the two video intervention groups RN-EXP and MP-EXP (*b* = 1.711, *SE* = 2.148, 95% CI = [-2.52, 5.94], *p = .*427). For further details, see **Figure 2**.

Results of the sensitivity analysis including multivariate outliers revealed a different pattern of results, showing no significant Time × Condition interaction (*F*(2,126) = 2.276, *p* = .107, *ω^2^* = .02).

Sensitivity analyses including prior treatment experience as an additional binary covariate showed that the pattern of results for treatment expectations remained unchanged. The Time × Condition interaction remained significant; *F*(2, 120) = 3.076, *p* = .049, *ω^2^* = .03, with a significant combined video- *vs*. -control contrast and no significant difference between the RN-EXP and MP-EXP conditions. Prior treatment experience was significantly associated with treatment expectations, *F*(1, 119) = 11.950, *p* <.001.

In the modified intention-to-treat sensitivity analysis including all participants with available pre- and post-intervention data regardless of task engagement, the Time × Condition interaction for treatment expectations was attenuated and no longer reached statistical significance (*F*(2,162) = 2.999, *p* = .052, *ω^2^* = .02). Planned contrasts were therefore not interpreted for this sensitivity analysis.

#### 3.3.2 Subjective treatment-seeking probability

**Figure 3:**
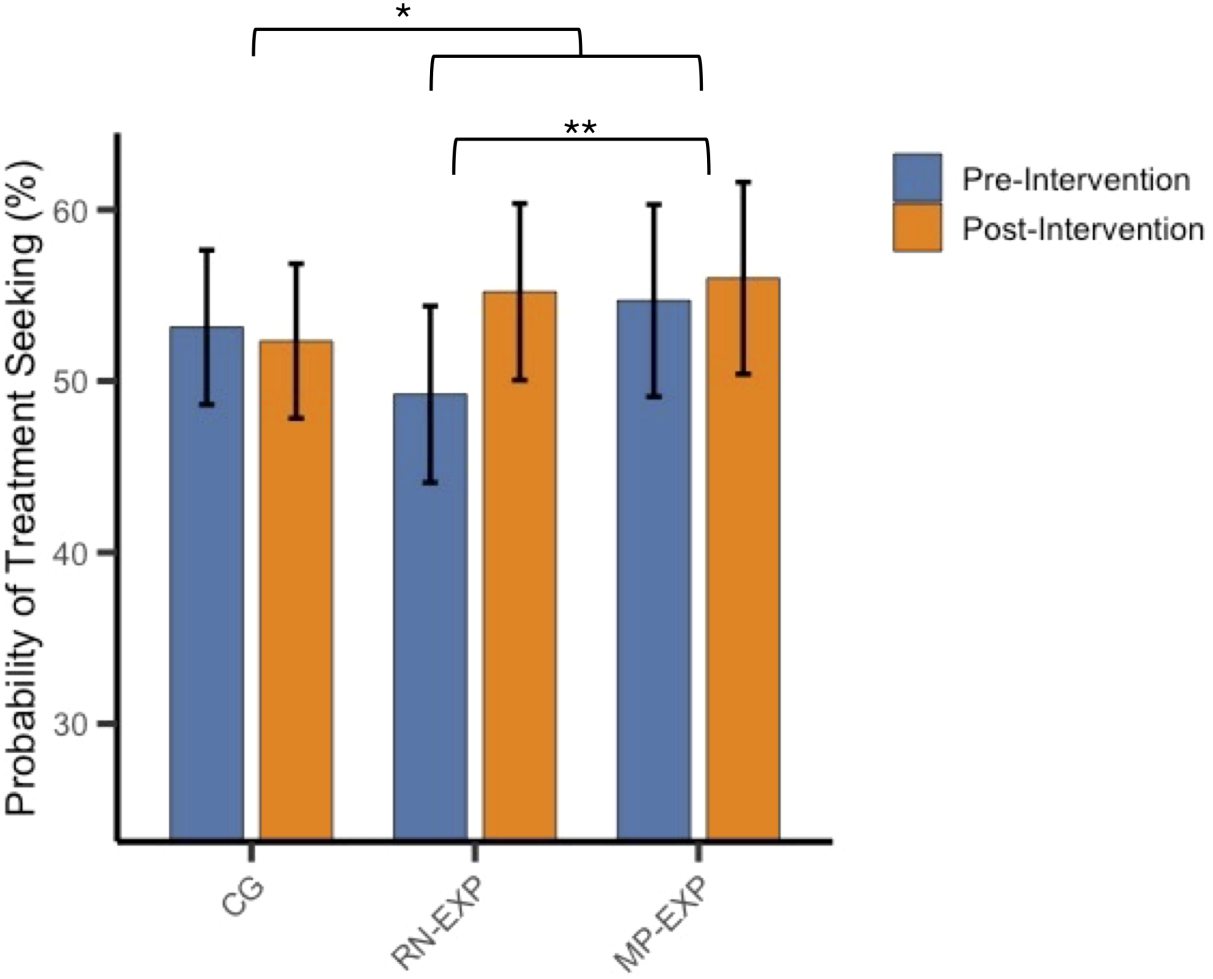
Change in treatment-seeking probability. Planned contrasts showed that participants in both video intervention groups (RN-EXP and MP-EXP; patient-therapist interaction) reported a larger increase in self-rated treatment-seeking probability (0-100%) than the minimal-intervention control group. In addition, RN-EXP showed a significantly larger increase than MP-EXP. Note. Error bars indicate standard errors. Descriptive values are reported as M (SD): RN-EXP pre = 49.23 (29.88), post = 55.21 (29.91); MP-EXP pre = 54.70 (35.16), post = 56.00 (34.88); CG pre = 53.14 (31.45), post = 52.33 (32.51). CG = minimal-intervention control group (brief writing task about treatment expectations); RN-EXP video intervention with avoidance-oriented framing to reduce negative treatment expectations; MP-EXP = video intervention with approach-oriented framing to increase positive treatment expectations. *p <= 0.05 , **p <= 0.01.

Mixed-effects ANOVA analyses indicated that participants reported a higher subjective likelihood of seeking psychological treatment after the intervention phase and that changes in subjective treatment-seeking probability differed significantly between experimental groups (Time × Condition: *F*(2,120) = 5.779, *p* = .004, *ω^2^* = .07). Planned contrasts indicated that, as expected, participants in both video intervention groups reported a significantly greater increase in subjective treatment-seeking probability after the intervention compared to the control group (*b* = 4.443, *SE* = 1.732, 95% CI = [1.03, 7.85], *p = .*017, *ω^2^* = .04). Furthermore, participants of the RN-EXP condition reported a higher subjective likelihood of seeking psychotherapy after intervention than those in the MP-EXP condition (*b* = 4.671, *SE* = 2.235, 95% CI = [0.27, 9.07], *p* = .039, *ω^2^* = .03). For more detailed information, see **Figure 3**.

Results of the sensitivity analyses showed no change in the pattern of results for subjective treatment-seeking probability when multivariate outliers were retained or when prior treatment experience was included as an additional binary covariate. Prior treatment experience was significantly associated with overall levels of subjective treatment-seeking probability, *F*(1, 119) = 5.022, *p* = .027.

In the modified intention-to-treat sensitivity analysis including all participants with available pre- and post-intervention data regardless of task engagement, the Time × Condition interaction for subjective treatment-seeking probability remained significant, *F*(2, 162) = 5.964, *p* = .003, *ω^2^* = .06. Planned contrasts indicated that the combined video intervention conditions showed a descriptively stronger increase compared with the control condition; however, this contrast no longer reached statistical significance (*b* = 4.137, *SE* = 2.130, 95% CI = [-0.05, 8.33], *p* = .054, *ω^2^* = .02). In contrast, the difference between the two video conditions remained significant, indicating a stronger increase in the RN-EXP condition compared with the MP-EXP condition (*b* = 6.976, *SE* = 2.460, 95% CI = [2.14, 11.82], *p* = .005, *ω^2^* = .04).

#### 3.3.3 Interest in treatment information

A Chi-squared test revealed no significant difference in interest in receiving treatment information between experimental groups (*χ²(*2, 123*)* = 0.607, *p* = .738, Cramér’s *V* = .07.).

#### 3.3.4 Secondary analysis

A secondary analysis was conducted using a subsample of participants who scored 35 or higher on the LSAS-SR (*n* = 111) as scores below 35 have been proposed as a remission threshold for social anxiety symptoms in a German sample (43). Results of the ANOVAs indicated that the experimental manipulation via video intervention did not significantly alter treatment expectations in this subsample when compared to the control condition (Time × Condition: *F*(2,108) = 2.159, *p* = .120, *ω^2^* = .02). However, the previously reported effects of the video intervention on subjective treatment-seeking probability were replicated. Participants with higher self-reported social anxiety who watched a video specifically targeting treatment expectations reported a higher subjective treatment-seeking probability than participants in the control condition (*b* = 5.046, *SE* = 1.732, 95% CI = [1.63, 8.46], *p = .*004, *ω^2^* = .06). *Post-hoc* contrast analyses further revealed that, similar to the results in the full sample, participants benefited more from the video framing designed to reduce negative treatment expectations than from the condition designed to maximize positive expectations (*b* = 4.763, *SE* = 2.184, 95% CI = [0.46, 9.06], *p = .*031, *ω^2^* = .03).

#### 3.3.5 Follow-up assessment: self-reported treatment-seeking behavior

Of the 123 participants included in the main analyses, 35 completed the one-week follow-up assessment, and 26 responses could be reliably linked to baseline and post-intervention data. Due to the small, linked sample and very small group sizes, follow-up data are reported descriptively only. Descriptive results for self-reported treatment-seeking steps at follow-up are presented in **Table 2**.

**Table 2:** Descriptive statistics for self-reported treatment-seeking steps in the linked follow-up sample.

Variable	RN (*N* = 6)	MP (*N* = 9)	CG (*N* = 11)
Treatment-seeking within the last week, *N (%)*			
Sought more information about treatment Visited online forums or online self-help groups Used an online therapy service Contacted an outpatient therapist or clinic	3 (50) 1 (16.67) 0 (0) 0 (0)	5 (55.56) 1 (11.11) 0 (0) 0 (0)	5 (45.45) 1 (9.10) 0 (0) 2 (18.18)

Values are based on the linked follow-up sample only (n = 26). Multiple responses were possible. Results are reported descriptively due to substantial attrition and very small group sizes. N, Number of participants; %, Percentage of participants.

## 4 Discussion

### 4.1 Summary and interpretation of results

The main finding of this study is that a brief role-model video designed to either reduce negative expectations or maximize positive expectations increased participants’ subjective likelihood of seeking psychotherapy compared to a control condition in which individuals described their own expectations. Furthermore, avoidance-oriented framing was more effective than approach-oriented framing in increasing subjective treatment-seeking probability. This pattern was replicated in a secondary analysis including participants with higher self-reported levels of social anxiety. Together, these findings suggest that even brief, expectation-focused interventions may support psychotherapy initiation by targeting expectation-related barriers.

These findings align with previous role-model-based interventions (44) and can be further understood from a social-cognitive perspective, reflecting processes of observational learning (45). Observing a person who initiates psychotherapy despite initial concerns and reports a positive treatment experience may increase the likelihood of seeking help. Such role models may influence expectations and perceived self-efficacy, thereby promoting more favorable attitudes toward help-seeking. In line with the health-psychology framework introduced above, the intervention may have affected expectation-related perceived benefits and barriers that are relevant for early psychotherapy engagement. The present study extends these findings by directly comparing two different framing approaches, with the avoidance-oriented framing emerging as more effective for increasing subjective treatment-seeking probability. This suggests that directly addressing expectation-related barriers such as concerns about burden, risks or negative outcomes may be particularly relevant for promoting help-seeking. In line with research on social anxiety, individuals with higher levels of self-reported social anxiety may be particularly responsive to such messages, as they tend to exhibit avoidance-oriented processing styles and heightened sensitivity to potential threats (46, 47). These individuals may experience stronger expectation-related barriers, making them particularly responsive to interventions that directly target such concerns.

Primary analyses suggested beneficial effects of both video interventions on treatment expectations; however, these findings were not robust across all sensitivity analyses and should therefore be interpreted with caution. Sensitivity analyses further indicated that prior treatment experience was associated with both treatment expectations and subjective treatment-seeking probability. However, including prior treatment experience as an additional binary covariate did not change the overall pattern of results for either outcome. Thus, prior treatment experience was related to overall outcome levels but did not account for the observed intervention effects. Treatment expectations were assessed with the Treatment Expectation Questionnaire (TEX-Q), a multidimensional instrument that covers several domains (benefit, positive impact, adverse effects, negative impact, process, behavioral control) and has demonstrated good psychometric properties (35, 48), making it unlikely that the lack of robust effects is due to a narrow or unreliable measure. At the same time, the TEX-Q is an explicit self-report scale and may be less sensitive to subtle, short-term changes in expectations and may be unable to capture more implicit components of treatment expectations (49). In addition, the control condition may have functioned as a low-intensity intervention. Research on mere-measurement and question–behavior effects suggests that reflecting on one’s expectations can itself influence subsequent perceptions and behavior (50–52), which may have reduced between-group differences. Finally, brief role-model videos may exert stronger effects on general motivation to seek treatment than on more specific treatment expectations and may therefore be particularly useful for addressing general psychological barriers to help-seeking. This is consistent with previous video-based interventions, which primarily improved attitudes and expectations toward psychotherapy at a more global level (44, 53, 54). Overall, effects on subjective treatment-seeking probability were more robust than effects on treatment expectations, with a similar pattern observed in the subgroup with higher self-reported social anxiety. However, sensitivity analyses showed a more differentiated pattern. In the modified intention-to-treat analysis, the overall Time × Condition interaction and the RN-EXP *vs*. MP-EXP contrast for subjective treatment-seeking probability remained significant, whereas the combined video-*vs*.-control contrast was attenuated and no longer reached statistical significance. Thus, the specific advantage of avoidance-oriented over approach-oriented framing appears more robust than the general effect of the video interventions compared with the control condition.

To further contextualize the link between treatment expectations and subjective treatment-seeking probability, exploratory correlational analyses were conducted. Exploratory analyses indicated small to moderate correlations between treatment expectation subscales and subjective treatment-seeking probability (*r* = .22–.32), with slightly stronger correlations for positive expectation subscales (*r* = .24-.37, *ps* <.01). However, these associations were modest and should be interpreted with caution. At the same time, these findings are consistent with the framework proposed by Griffiths (17), in which treatment expectations are only one of several factors influencing help-seeking behavior, alongside structural barriers, stigma, symptom severity and social support.

No significant differences were found between experimental groups regarding interest in receiving more specific treatment information. One possible explanation is that this outcome represented a very low-threshold behavioral indicator, which may have resulted in ceiling effects and reduced sensitivity to detect group differences. However, it may also indicate that changes in subjective treatment-seeking probability did not translate into immediate behavioral steps, consistent with research on the intention-behavior gap (24). Accordingly, subjective treatment-seeking probability should be interpreted as an indicator of help-seeking intention rather than actual treatment-seeking behavior. The follow-up data were too limited to determine whether these changes translated into subsequent behavior.

### 4.2 Limitations

The present study has several limitations that should be considered when interpreting the findings.

First, the two video micro-interventions differed only in subtle aspects of expectation framing (avoidance- *vs*. approach-oriented), while otherwise being highly similar in content and structure. Although this allowed us to isolate specific framing effects, it also resulted in relatively small between-group differences. Task engagement was assessed after the intervention, including whether participants watched the video attentively, felt distracted and were able to follow the conversation. However, the study did not include a framing-specific manipulation check. Thus, these items provided information about engagement with the intervention material but did not directly assess whether participants explicitly perceived the intended avoidance- versus approach-oriented framing difference. In addition, no condition combining both framing approaches was included, leaving open the question of whether an integrated strategy might yield a stronger effect.

Second, the interventions were implemented as standardized role-play videos using doctoral students from the research group as patient and therapist, following a fixed script. While this procedure ensured a high level of experimental control, it limits ecological validity and variability in patient–therapist interactions. Both videos showed the same dyad (female patient, male therapist), although research on role models and social learning suggests that perceived similarity and identification with a model can influence intervention effects (45, 55–57). The fixed, scripted dyad may therefore have facilitated identification for some participants while limiting it for others and may reduce generalizability to routine care settings.

Third, the intervention and assessments were conducted entirely online and without supervision. The online format was chosen to reduce participation barriers for individuals with higher self-reported social anxiety levels and to allow for efficient data collection, but it also reduced control over contextual factors such as distractions, technical problems, and variations in attention and engagement. Although sensitivity analyses partly addressed this issue, differential task-engagement exclusions across conditions should be considered when interpreting the findings. Thus, the results may be most informative for participants who sufficiently engaged with the intervention material.

The outcome ‘interest in treatment information was measured as participants’ willingness to provide an email address to receive additional information about treatment options, which represents a broad but initial indicator of treatment-seeking behavior. The planned one-week follow-up could not be meaningfully analyzed due to substantial attrition and data linkage limitations. Although high dropout is common in internet-based interventions (58–60), the attrition observed here limits the interpretability of the behavioral follow-up data. As a result, the present findings primarily reflect short-term changes in expectations and help-seeking intentions, while conclusions about actual help-seeking behavior remain tentative.

Finally, the sample consisted predominantly of young, female, and highly educated participants. Such WEIRD samples (Western, Educated, Industrialized, Rich, Democratic) are common in early-stage research but limit generalizability to the broader population (61, 62). Although groups did not differ significantly in self-reported current mental disorder status or prior treatment experience, these variables were assessed only broadly and by self-report. Unmeasured differences in comorbid mental health conditions or in participants’ stage of help-seeking may therefore still have influenced treatment expectations and intervention responses.

### 4.3 Future perspectives

Future research should examine framing effects not only over brief intervals, but across longer time periods and ideally throughout the course of psychotherapy. Many psychotherapeutic approaches aim to shift patients from avoidance-oriented to approach-oriented goals (63–65). Avoidance-oriented framing may be particularly useful before or at the beginning of psychotherapy by addressing expectation-related barriers, whereas approach-oriented framing may become more relevant as therapy progresses. Longitudinal designs could clarify whether the short-term effects observed in the present study translate into sustained changes in expectations and actual treatment engagement.

From a practical perspective, the findings do not suggest that the specific video format should be directly implemented in routine care. Rather, they suggest that the way psychotherapy-related information is framed may be relevant, especially before treatment begins. Avoidance-oriented wording that emphasizes reduced burden and manageability of treatment-related concerns could be used at early points of contact. This may include clinic websites, psychoeducational materials or initial consultations. Such communication could be provided by mental health services, outpatient clinics, counselling centers, or therapists during early contact with potential patients. Future studies should test whether these framing strategies are feasible, acceptable, cost-efficient, and effective in routine clinical pathways.

In addition, future studies should compare the mechanisms underlying role-model videos and text-based information. Studies could contrast video-based role models with equivalent text-based information and combined formats. This could clarify whether observed effects are primarily driven by observational learning and identification with the model or by the informational content of the framing itself. Further, implicit measurement methods such as the Therapy Single Category Implicit Association Test (Therapy SC-IAT; 49) could be used to assess implicit expectations and compare them with self-reports. This would make it possible to investigate whether changes in framing differentially affect explicit and implicit components of treatment expectations.

Moreover, prior treatment experiences should be considered more systematically. Previous experiences may shape expectations and influence how individuals respond to different framing approaches (66, 67). Although prior treatment experience was assessed and included in sensitivity analyses in the present study, it was measured only as a broad binary indicator of previous treatment use. Future studies should assess the timing, quality and perceived helpfulness of prior treatment experiences and examine whether positive, negative or ambivalent treatment experiences moderate responses to avoidance- versus approach-oriented framing.

The present findings should be replicated in well-characterized clinical samples, as the current study relied on self-reported symptom levels, which provide only a limited indication of clinical diagnoses. Beyond social anxiety, similar framing effects should also be examined in other clinical populations such as anxiety and mood disorders, obsessive–compulsive and somatoform disorders, chronic pain, or psychotic disorders, as expectations and barriers to treatment may differ substantially across groups.

Qualitative methods, such as semi-structured interviews or open-ended responses, could further improve understanding of expectation-related barriers and decision-making processes related to help-seeking. These insights could support the development of more tailored, group-specific and role-model-based interventions.

## 5 Conclusions

This study suggests that specific expectation-focused framing can increase the short-term, self-reported likelihood of seeking psychotherapy. In particular, avoidance-oriented framing, which emphasized relief and reduced burden, was more effective than approach-oriented framing in increasing subjective treatment-seeking probability, both in the total sample and in participants with higher self-reported social anxiety. These findings highlight the importance of targeting expectation-related barriers to psychotherapy initiation.

The results point to the importance of how psychotherapy-related information is communicated before treatment begins. Expectation-focused communication may be delivered through different formats, such as wording in written materials, information provided during first contact, online resources or brief role-model-based videos. Applying different framing strategies at different stages of the help-seeking process may further enhance their effects.

At the same time, expectations are only one of several determinants of psychotherapy uptake, and structural barriers and stigma remain important challenges. Nonetheless, expectation-related processes represent a modifiable target that can be addressed through communication strategies in clinical settings, particularly before and during the early phase of psychotherapy. Future research should replicate these findings in larger and more diverse samples and examine how expectation-focused communication can be implemented in routine care.

## Data Availability

The datasets presented in this article are not readily available because of ethical restrictions and participant privacy concerns. De-identified data may be made available by the corresponding author upon reasonable request, provided that data sharing is consistent with participants’ informed consent and applicable data protection regulations. Requests to access the datasets should be directed to leonora.schaefer@uni-marburg.de.
